# Inhibitory effects of *Nigella sativa* seed oil on the testosterone-induced benign prostatic hyperplasia in rats

**DOI:** 10.37796/2211-8039.1083

**Published:** 2021-03-01

**Authors:** Arezo Sadeghimanesh, Sajedeh Gholipour, Akram Torki, Hossein Amini-khoei, Zahra Lorigooini, Solomon Habtemariam

**Affiliations:** a Medical Plants Research Center, Basic Health Sciences Institute, Shahrekord University of Medical Sciences, Shahrekord, Iran; b Pharmacognosy Research Laboratories and Herbal Analysis Services UK, University of Greenwich, Chatham-Maritime, Kent, ME4 4TB, UK

**Keywords:** antioxidant level, benign prostatic hyperplasia, dihydrotestosterone, malondialdehyde, *Nigella sativa* seed oil, prostate-specific antigen

## Abstract

**Background:**

Benign prostatic hyperplasia (BPH) is the most prevalent disease of the prostate in elderly men. Since *Nigella sativa* has been reported to show various pharmacological effects, this study was conducted to examine the effect of *N*. *sativa* seed oil on experimental BPH.

**Methods:**

The oil was extracted using the cold-pressing method. Fifty rats were divided into five groups of 10 each as follows: Group 1 orally (p.o.) received normal saline; groups 2–5 were castrated and subcutaneously received 5 mg/kg testosterone propionate for four weeks. Group 2, namely, BPH model, underwent no further treatment, Groups 3 and 4 were treated with 400 mg/kg and 800 mg/kg *N. sativa* seed oil, Group 5 received finasteride (0.5 mg/kg, p.o.) for 28 days. All groups received repeated testosterone injections for the following four weeks after BPH induction. After the treatments, rats were sacrificed and the prostate tissues removed. Wet weight, prostatic volume (PV) and prostatic index (PI) were determined. Serum prostate-specific antigen (PSA), dihydrotestosterone (DHT), malondialdehyde (MDA) and antioxidant levels were determined.

**Results:**

Our results showed that oral treatment with 400 and 800 mg/kg *N. sativa* oil led to a significant decrease in PI, PV, DHT concentration, PSA, and serum MDA level, and also significantly increased serum antioxidant capacity.

**Conclusions:**

The study demonstrated that the oil seed exerted anti-BPH effects which may be associated with its antioxidant properties *in vivo*.

## 1. Background

Benign prostatic hyperplasia (BPH) is the most prevalent age-related disease of the prostate gland for men [1,2]. Its symptoms include urinary tract obstruction, frequent urination, urinary retention, decreased diameter of the urinary tube and pressure of urine flow, and dribbling at the end of urination [3,4]. The disease is characterized by prostate gland enlargement due to hyperproliferation of cellular components such as mesenchymal cells. The most common drug treatments for BPH include the use of α-adrenergic antagonists, 5-α-reductase inhibitors and alternative therapies such as natural products [5].

Recent studies have shown the relationship between oxidative stress (OS) and BPH. As a measure of OS, the level of the lipid peroxidation indicator, malondialdehyde (MDA) increases in BPH patients while plasma antioxidants’ are suppressed [6,7]. This implies that antioxidant therapies might have potential application in the management of BPH.

Alpha-adrenergic receptor blockers and 5-alpha reductase inhibitors cause side effects, and the high prices of some of these drugs have led to an increasing tendency to use natural compounds as a source of lead compounds for drug design for the treatment of BPH or as a supplementary drug. Studies showed that alternative and complementary treatment is remarkable options for the management of mild BPH patients including *Serona repen*, *Pygeum africanum*, and *Secale cereal*. There are several reasons for significant attitude for this global approach including their availability, low cost as well as showing better safety profile than the current pharmaceutical medications. Furthermore, due to the universal approach to returning to nature and the use of natural compounds in the treatment of incurable diseases, attention to plants along with other natural resources has increased, among these, it could mention to *Nigella sativa* that it is highly recommended by the researches [5].

Belonging to the plant family of Ranunculaceae, *Nigella sativa* is native to southwestern Asia, southern Europe, northern Africa and Iran [8,9]. Mainly cultivated for its black seeds, *N. sativa* has extensive applications as a spice and medicinal plant. The seeds are also rich sources of fixed oil, which is renowned for a high level of unsaturated fatty acids such as oleic, linoleic and linolenic acid. Previous studies have further reported that these fatty acids can prevent the proliferation of prostate cells induced by testosterone and DHT. Additionally, they are capable of inhibiting the 5-α-reductase, an enzyme drug target of DHT which is known to metabolise testosterone to dihydrotestosterone [10–12]. *N*. *sativa* has further been demonstrated to exert antioxidant and anti-inflammatory properties [13,14]. No evidence has yet been reported, however, on the use of this plant to treat BPH. This study was thus conducted to evaluate the effect of *N*. *sativa* seed oil on rat model of BPH.

## 2. Materials and methods

### 2.1. Preparation of N. sativa seeds and oil extraction

Cultivated *N*. *sativa* seeds were purchased from Shahrekord Agricultural Faculty, Shahrekord, Iran. Samples were cultivated in the region of (32° 21′ 00′′ North, 50° 49′ 00′′ East) where the average rainfall from cultivation to harvest is reportedly 5–7 mm. A voucher specimen (SKUMS-801) was approved by a botanical expert (Shirmardi, Hamzeh Ali, PhD. at the Iranian Research Center of Agriculture and Natural Resources, P.O. Box 415, Shahrekord) and deposited in the Herbarium of Medical Plants Research Center affiliated to Shahrekord University of Medical Sciences. The seeds were kept in polyethene bags at 4 °C and then the dried seeds were extracted using screw less cold presses machine. The seeds were pressed at 50 °C with nozzle size 7 mm and speed of screw at 20 rpm. The crude oil gained was kept in an amber bottle to store in the freezer (−18 °C) until the next analysis [15].

### 2.2. Husbandry

Fifty male Wistar rats weighing between 200 and 250 g were obtained from the Pasteur Institute of Iran (Tehran, Iran). The animals were housed under 21–23 °C temperature and 12-h light and 12-h darkness cycles for seven days to acclimatize to the animal house. All stages of experimentation were carried out under the regulations of the ethic committee of shahrekord university of medical sciences (ethics code: IR.SKUMS.REC.1394.28).

### 2.3. Experimental design

#### 2.3.1. Castration and testosterone-induced rat model of BPH

**Fig. 1:**
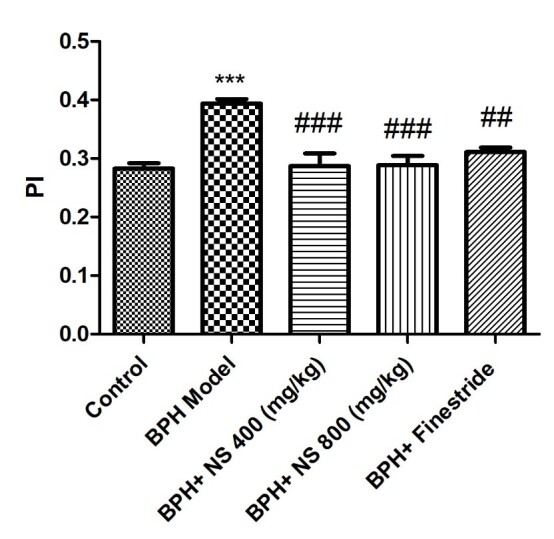
Schematic of study design.

First, the rats were anaesthetized using 50 mg/kg phenobarbital and their testes were removed under sterile conditions. After castration, penicillin (7.14 × 104 IU/kg body weight) was administrated intramuscularly according to a previously described method [2]. Seven days later, the animals subcutaneously received 5 mg/kg testosterone propionate daily for four weeks. Simultaneously, group 1, as negative controls, were administered with normal saline alone, group 2 were considered BPH model, groups 3 and 4 were orally treated with 400 and 800 mg/kg of the oil of *N*. *sativa* seed, respectively, group 5 were administered with 0.5 mg/kg oral finasteride and considered positive control [16]. The doses of *N. sativa* seed oil were selected according to previous studies [17,18]. All groups received repeated testosterone injections for the following four weeks after BPH induction (Fig. 1). After 28 days, the rats fasted for 24 h and blood samples were drawn from the abdominal aorta under deep anaesthesia. The prostate glands were then removed for further examinations. At the end of the experiments, rats were euthanized using a high dose of co-administered ketamine and xylazine.

#### 2.3.2. Determination of prostate index (PI) and volume (PV)

After anaesthesia, the whole prostates were collected and immediately weighed. The prostate weight/total body weight ratio was considered to indicate PI [19] and the immersion of prostate in graded acetone was measured to indicate PV [20].

#### 2.3.3. Measurement of prostate-specific antigen (PSA)

The PSA was measured by Biotin double antibody sandwich method utilizing an ELISA kit (Shanghaicristal Day Biotech Co., China) according to the manufacturer’s instructions.

#### 2.3.4. Determination of dihydrotestosterone (DHT)

DHT was measured by mean of standard ELISA kits (Shanghaicristal Day Biotech Co., China) according to the manufacturer’s instructions (Shanghaicristal Day Biotech Co., China). 10 μl of standard control and serum sample was poured into the plate and 50 μl biotin and 50 μl conjugate were added to them and incubated for 1 h at room temperature and then the plate was washed with a washing machine (Chrom, Asrs Atlantis Washer) and 100 μl of the substrate with the dye solution added to it and for the sample concentration determination was placed inside the Elisa reader. According to the OD standards, the diagram was drawn and the concentration of the samples in ng/ml was determined according to the diagram. From control serum was used for quality control of the kit and to ensure the accuracy of the test result, the concentration of control serum was considered in the range defined by the kit.

#### 2.3.5. Determination antioxidant capacity of the serum

Blood samples were collected from all animals using cardiac puncture, and the serum was separated by centrifugation. The Ferric Reducing Ability of Plasma (FRAP) assay was applied for measuring the total serum antioxidant capacity. This method is based on the ability of the serum to reduce ferrictripiridyltriazine (Fe^3+^-TPTZ) to a ferrous form (Fe^2+^), yielding a blue coloured complex (Fe^2+^-TPTZ) with maximum optical absorbance at 593 nm [21].

#### 2.3.6. Determination of serum MDA levels

For measuring serum MDA level, 0.5 g of thiobarbituric acid was dissolved in 80 ml 20% acetic acid and then the pH of the solution was set at 3.5 by using NaOH. The final volume of the assay was then adjusted to 100 ml by addition of 20% acetic acid. Then, 100 μl of the serum sample was dissolved in 2.5 ml of the working solution and 100 μl of 8.1% sodium dodecyl sulfate (SDS). The samples were left in a water bath containing boiling water for 1 h and then cooled and centrifuged at 4000 rpm. The supernatant’s optical absorbance was read at a wavelength of 523 nm [22].

### 2.4. Statistical analysis

The data were presented as mean ± standard error of measurement. Data analysis was performed by one-way ANOVA and Tukey’s test using version 7 of the GraphPad Prism software. Data were considered statistically significant at the level of *P* < 0.05.

## 3. Results

### 3.1. The effect of N. sativa seed oil on PI and PV

**Fig. 2:**
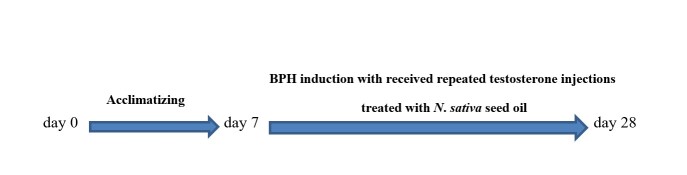
Effect of Nigella sativa seed oil on prostate index; control: Healthy rats, BPH model: Rats with BPH, BPH+ NS 400 (mg/kg): Rats with BPH treated with 400 mg/kg N. sativa seed oil, BPH+ NS 800 (mg/kg): Rats with BPH treated with 800 mg/kg N. sativa seed oil, BPH+Finestride: Rats with BPH administered with 0.5 mg/kg finasteride; *** significant difference with control group ( p < 0.001), ###,###,## significant difference with BPH group ( p < 0.001, p < 0.001, p < 0.01).

**Fig. 3:**
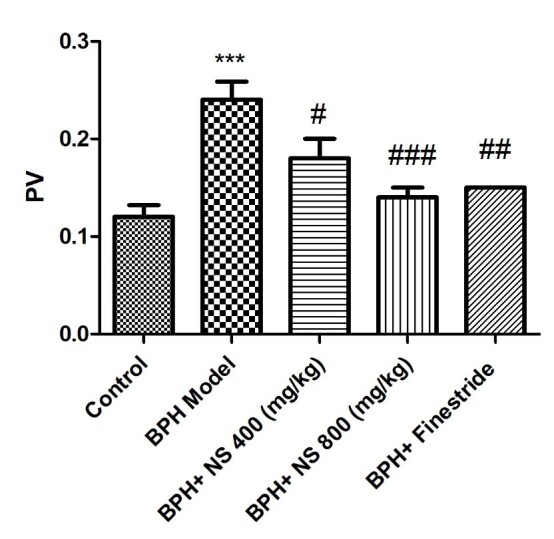
Effect of Nigella sativa seed oil on prostate volume; control: Healthy rats, BPH model: Rats with BPH, BPH+ NS 400 (mg/kg): Rats with BPH treated with 400 mg/kg N. sativa seed oil, BPH+ NS 800 (mg/kg): Rats with BPH treated with 800 mg/kg N. sativa seed oil, BPH+Finestride: Rats with BPH administered with 0.5 mg/kg finasteride; *** significant difference with control group ( p < 0.001), #, ###, ## significant difference with BPH model group ( p < 0.05, p < 0.001, p < 001).

As Figs. 2 and 3 illustrate, the highest PI and PV are observed in the BPH model group and the lowest PI and PV levels in the control group ( *p* < 0.001). Oral treatment with 400 and 800 mg/kg of the oil of *N*. *sativa* seed and finasteride significantly decreased the PI and PV when compared to the BPH model group ( *p* < 0.05).

### 3.2. The effect of N. sativa seed oil on DHT and PSA concentrations

**Fig. 4:**
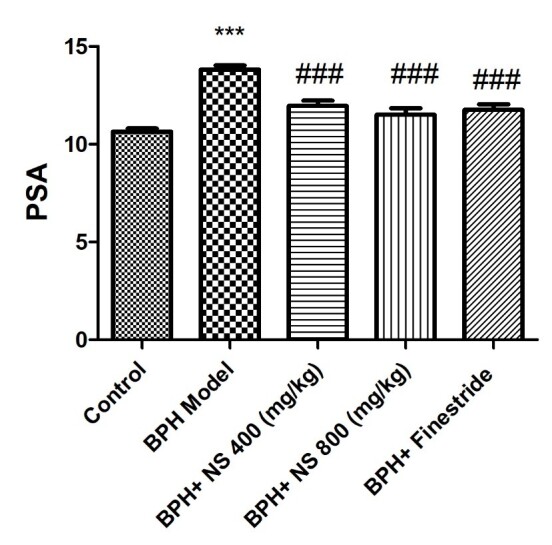
Effect of Nigella sativa seed oil on dihydrotestosterone concentration; control: Healthy rats, BPH model: Rats with BPH, BPH+ NS 400 (mg/kg): Rats with BPH treated with 400 mg/kg N. sativa seed oil, BPH+ NS 800 (mg/kg): Rats with BPH treated with 800 mg/kg N. sativa seed oil, BPH+Finestride: Rats with BPH administered with 0.5 mg/kg finasteride; *** significant difference with control group ( p < 0.001), ### significant difference with BPH model group ( p < 0.001).

**Fig. 5:**
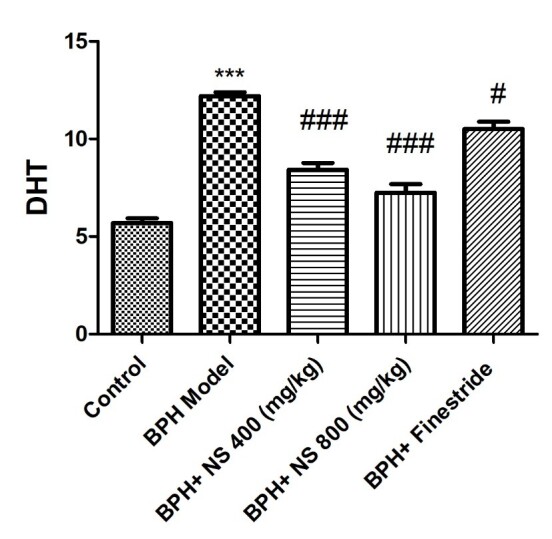
Effect of Nigella sativa seed oil on prostate-specific antigen concentration; control: Healthy rats, BPH model: Rats with BPH, BPH+ NS 400 (mg/kg): Rats with BPH treated with 400 mg/kg N. sativa seed oil, BPH+ NS 800 (mg/kg): Rats with BPH treated with 800 mg/kg N. sativa seed oil, BPH+Finestride: Rats with BPH administered with 0.5 mg/kg finasteride; *** significant difference with control group ( p < 0.001), ###, ###, # significant difference with BPH model group ( p < 0.001, p < 0.001, p < 0.05).

As shown in Figs. 4 and 5, the highest concentrations of DHT and PSA were observed in the BPH model group while the lowest DHT and PSA concentrations were evident in the control group ( *p* < 0.001). Oral treatment with 400 and 800 mg/kg of the oil of *N*. *sativa* seed and finasteride significantly decreased the DHT and PSA concentrations compared to the BPH model group ( *p* < 0.05). The level of DHT also decreased significantly and more markedly in both *N. sativa* seed oil-treated groups when compared to the finasteride-treated group (p < 0.05).

### 3.3. The effect of N. sativa seed oil on serum antioxidant capacity and MDA levels

**Fig. 6:**
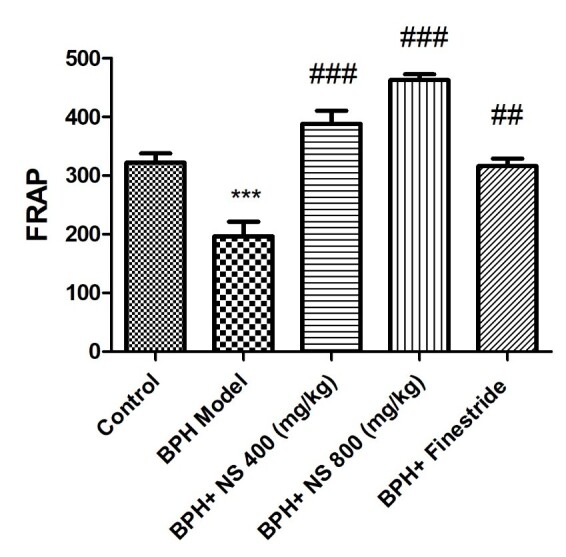
Effect of Nigella sativa seed oil on serum antioxidant capacity; control: Healthy rats, BPH model: Rats with BPH, BPH+ NS 400 (mg/kg): Rats with BPH treated with 400 mg/kg N. sativa seed oil, BPH+ NS 800 (mg/kg): Rats with BPH treated with 800 mg/kg N. sativa seed oil, BPH+Finestride: Rats with BPH administered with 0.5 mg/kg finasteride; *** significant difference with control group ( p < 0.001), ###, ###, ## significant difference with BPH model group ( p < 0.001, p < 0.001, p < 0.01).

As Fig. 6 illustrates the lowest serum antioxidant capacity level is observed in the BPH model group and the highest serum antioxidant capacity level in the control group ( *p* < 0.001). Treatment with 400 and 800 mg/kg N*. sativa* seed oil and finasteride significantly increased the serum antioxidant capacity level concentration in comparison to the BPH model group ( *p* < 0.05). The results showed that treatment with 800 mg/kg of the oil of *N. sativa* seed significantly increased the serum antioxidant capacity when compared to the finasteride receiving group ( *p* < 0.001).

**Fig. 7:**
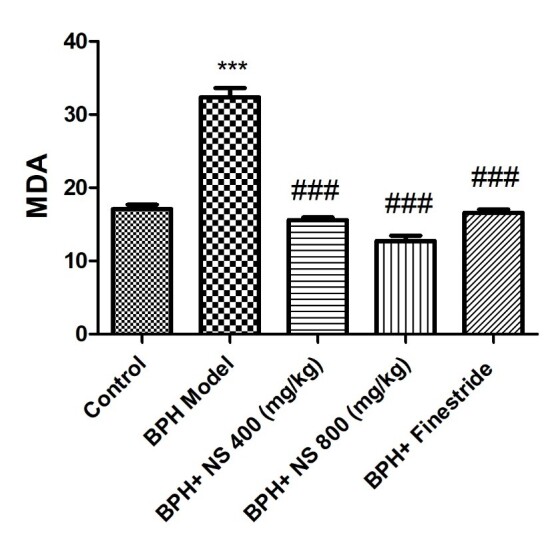
Effect of Nigella sativa seed oil on malondialdehyde concentration; control: Healthy rats, BPH model: Rats with BPH, BPH+ NS 400 (mg/kg): Rats with BPH treated with 400 mg/kg N. sativa seed oil, BPH+ NS 800 (mg/kg): Rats with BPH treated with 800 mg/kg N. sativa seed oil, BPH+Finestride: Rats with BPH administered with 0.5 mg/kg finasteride; *** significant difference between control group and other groups ( p < 0.001), ### significant difference with BPH model group ( p < 0.001).

Figure 7 illustrates the effect of oral treatment with *N*. *sativa* seed and finasteride oil on MDA concentration. the highest MDA level is observed in the BPH model group and the lowest MDA level in the control group ( *p* < 0.001). Treatment with 400 and 800 mg/kg N*. sativa* seed oil significantly decreased the MDA concentration in comparison to the BPH model group ( *p* < 0.001). Besides, 800 mg/kg N. *sativa* seed oil treatment led to a significant decrease in MDA concentration when compared to the finasteride-treated group ( *p* < 0.05).

## 4. Discussion

The BPH is a non-malignant growth of the epithelial and stromal cells of the prostate gland. 5α-Reductase inhibitors and alpha-1-adrenergic antagonists are two main agents commonly used to treat BPH. 5α-Reductase is an essential enzyme that converts testosterone to dihydrotestosterone [23–25].

Finasteride is a classical 5α-reductase inhibitor that decreases the DTH level, resulting in a decrease in the PV and symptoms of patients with BPH [26,27]. It has been well established that α_1_-adrenoreceptor blockers relax prostatic smooth muscles thereby increasing urine flow while decreasing the prostate size and PSA [28]. The beneficial effects of medicinal plants in treating BPH have already been confirmed [8]. It has been well established that medicinal plants used to treat BPH decrease the plasma and prostate levels of the DHT and consequently suppress prostate weight and size [29]. In the current study and consistent with previous studies [3,26,28,30], we observed that induction of BPH led to increasing in PSA, DHT, PV, and PI in a rat model. Besides that, our findings showed that *N*. *sativa* seed oil treatment significantly mitigated these pathological markers that both doses of the 400 and 800 mg/kg showed this effective effect.

In the current study, treatment with 400 and 800 mg/kg of the oil of *N*. *sativa* seed decreased the DHT level. “Interestingly, we found that the *N*. *sativa* oil partially at least decreased the DHT level in the BPH model” more than that of finasteride. Hiipakka et al. reported that treatment with polyphenols isolated from green tea decreased the DHT production, and inhibited prostate cells proliferation. Because *N*. *sativa* contains polyphenolic compounds [31], it can be argued that, at least, inhibition of 5α-reductase contributes to the beneficial effect of this plant. The high amount of fatty acids of *N*. *sativa* oil has essential unsaturated fatty acids (about 1% omega-3, 25% omega-9 and 58% omega-6) in abundance [10,12]. Abdel-Rahman et al. have argued that compounds rich in fatty acids could prevent prostate cells proliferation by lowering testosterone and DHT concentrations [9]. Liang et al. further demonstrated that fatty acids could inhibit 5α-reductase [10]. It has been shown that increased prostate weight could be considered a marker to diagnose BPH, while PI is often used to determine the progression of BPH [20]. In the present study, both 400 and 800 mg/kg doses of the oil of *N*. *sativa* seed significantly decreased the PI and PV in BPH.

Recently, it has been demonstrated that PV and PSA concentrations can be used to predict prostate cells growth. In this regard, PSA can be considered as an alternative index for PV and as a marker to detect the risk of prostate carcinoma [20,32]. Hence, an increased PSA level represents an increased proliferation of prostate cells. In the current study, treatment with 400 and 800 mg/kg N. *sativa* seed oil significantly decreased the PSA concentration compared to the BPH model group. Ren et al. reported that polyphenols can suppress the level of expression of PSA genes [33]. *N. sativa*’s effect in decreasing the PSA may thus be related to the presence of polyphenols.

It has been determined that inflammation contributes to the pathophysiology of BPH because inflammatory factors such as monocyte chemotactic protein-1 are overexpressed. Hence the levels of interleukin 10 receptor subunit alpha (IL-10RA) and Interleukin 8 receptor, beta (IL-8RB) rise in the BPH [34]. According to the study by Ragheb et al., thymoquinone isolated from *N*. *sativa* has an anti-inflammatory property and can decrease the expression of the above-mentioned inflammatory factors. It seems that *N*. *sativa* oil’s effect can be to some extent attributed to the presence of thymoquinone in *N*. *sativa* and the anti-inflammatory property of this plant [35]. According to the study of Jonas et al., the antioxidant activities of plants help regulate cell proliferation and control in BPH [36]. It has been demonstrated that an increase in the level of MDA, which occurs in BPH, is a marker of lipid peroxidation and/or tissues damage. Increased MDA level in BPH has also been reported to be due to OS [6]. Hence, treatment with antioxidants may decrease the level of MDA and other pathological markers of BPH. In our study, treatment with 400 and 800 mg/kg of the oil of *N*. *sativa* seed decreased the serum MDA level, which is consistent with the study by Hosseinzadeh et al. [37].

Also, in our study, treatment with 400 and 800 mg/kg of the oil of *N*. *sativa* seed increased the serum antioxidant capacity level. Houcher reported that the oral treatment with *N*. *sativa* extract can cause an increase in the FRAP capacity [38], which is consistent with our results.

Therefore, it seems that *N*. *sativa* oil’s effects to inhibit lipid peroxidation and probably its anti-BPH effects are due to the presence of antioxidant and free radical-inhibiting compounds. The results on the serum MDA levels in the current study further demonstrated that these variables increased in the BPH group in comparison to the control group. In addition, the serum MDA levels decreased significantly in *N*. *sativa* oil-treated groups in comparison to the BPH group, which indicates the protective effects of the compounds present in the *N*. *sativa* oil could increase the antioxidant capacity of serum and decrease the level of MDA. It seems that this decrease in MDA levels is associated with the antioxidant capacity of the plant.

## 5. Conclusion

According to the current study, *N. sativa* seed oil in both doses of 400 and 800 mg/kg may have application in treating BPH by decreasing the concentrations of DHT and PSA, and PI and PV and exerting an antioxidant effect. Further studies are required to isolate and identify the active component( s) of the oil.

## Supplementary Information











## References

[1] Chen J Song H Protective potential of epigallocatechin-3-gallate against benign prostatic hyperplasia in metabolic syndrome rats Environ Toxicol Pharmacol 2016 45 315 20 27348728 10.1016/j.etap.2016.06.015

[2] Xu DH Wang LH Mei XT Li BJ Lu JL Xu SB Protective effects of seahorse extracts in a rat castration and testosterone-induced benign prostatic hyperplasia model and mouse oligospermatism model Environ Toxicol Pharmacol 2014 37 2 679 88 24607683 10.1016/j.etap.2014.02.001

[3] Afriyie DK Asare GA Bugyei K Adjei S Mao Lin J Peng I Treatment of benign prostatic hyperplasia with Croton membranaceus in an experimental animal model J Ethnopharmacol 2014 157 90 8 25256687 10.1016/j.jep.2014.09.007

[4] Simpson R Benign prostatic hyperplasia Br J Gen Pract 1997 47 235 40 9196969 PMC1312951

[5] Hansen BJ Hald T Review of current medical treatment of benign prostatic Hyperplasia Eur Urol 1993 24 41 9 7687558 10.1159/000474373

[6] Aryal M Pandeya A Gautam N Baral N Lamsal M Majhi S Oxidative stress in benign prostate hyperplasia Nepal Med College J 2007 9 222 4 18298008

[7] Merendino RA Salvo F Saija A Di Pasquale G Tomaino A Minciullo PL Malondialdehyde in benign prostate hypertrophy: a useful marker? Mediat Inflamm 2003 12 2 127 8 10.1080/0962935031000097745 PMC1781597 12775364

[8] Benkaci-Ali F Akloul R Boukenouche A Pauw ED Chemical composition of the essential oil of *Nigella sativa* seeds extracted by microwave steam distillation J Essent Oil Bearing Plant 2013 16 781 94

[9] Abdel-Rahman MK Effect of pumpkin seed (*Cucurbita pepo L*.) diets on benign prostatic hyperplasia (BPH): chemical and morphometric evaluation in rats World J Chem 2006 1 33 40

[10] Liang T Liao S Inhibition of steroid 5α-reductase by specific aliphatic unsaturated fatty acids Biochem J 1992 285 557 62 1637346 10.1042/bj2850557 PMC1132824

[11] Khare C Encyclopedia of Indian medicinal plants-rational Western therapy 3 Germany Ayurvedic and other Traditional Usage Springer 2004 540 20033

[12] Ahmad A Husain A Mujeeb M Khan Sh Najmi A Siddique A A review on therapeutic potential of *Nigella sativa*: a miracle herb Asian Pac J Trop Biomed 2013 3 337 52 23646296 10.1016/S2221-1691(13)60075-1 PMC3642442

[13] Abdel-Fattah A-FM Matsumoto K Watanabe H Anti-nociceptive effects of *Nigella sativa* oil and its major component, thymoquinone, in mice Eur J Pharmacol 2000 400 1 89 97 10913589 10.1016/s0014-2999(00)00340-x

[14] Khan MA Afzal M Chemical composition of *Nigella sativa*. Linn: Part Recent advances Inflammopharmacology 2016 24 67 79 27068721 10.1007/s10787-016-0262-7 PMC4883276

[15] Gharby S Harhar H Guillaume D Roudani A Boulbaroud S Ibrahimi M Chemical investigation of *Nigella sativa L*. seed oil produced in Morocco J Saudi Soc Agri Sci 2015 172 7

[16] Hieble JP Andersson KE Michel MC Animal models for benign prostatic hyperplasia Handb Exp Pharmacol 2011 2011 69 79 10.1007/978-3-642-16499-6_4 21290222

[17] De Lourdes Arruzazabala M Molina V Más R Carbajal D Marrero D González V Effects of coconut oil on testosterone-induced prostatic hyperplasia in Sprague-Dawley rats J Pharm Pharmacol 2007 59 995 9 17637195 10.1211/jpp.59.7.0012

[18] Tsai YS Tong YC Cheng JT Lee CH Yang FS Lee HY Pumpkin seed oil and phytosterol-F can block testosterone/prazosin-induced prostate growth in rats Urol Int 2006 77 269 74 17033217 10.1159/000094821

[19] Atawia RT Mosli HH Tadros MG Khalifa AE Mosli HA Abdel-Naim AB Modulatory effect of silymarin on inflammatory mediators in experimentally induced benign prostatic hyperplasia: emphasis on PTEN, HIF-1α, and NF-κB N Schmied Arch Pharmacol 2014 387 1131 40 10.1007/s00210-014-1040-y 25164963

[20] Roehrborn CG Boyle P Gould AL Waldstreicher J Serum prostate-specific antigen as a predictor of prostate volume in men with benign prostatic hyperplasia Adult Urol cme Article 1999 53 581 9 10.1016/s0090-4295(98)00655-4 10096388

[21] Benzie IF Strain J [2] Ferric reducing/antioxidant power assay: direct measure of total antioxidant activity of biological fluids and modified version for simultaneous measurement of total antioxidant power and ascorbic acid concentration Methods Enzymol 1999 299 15 27 9916193 10.1016/s0076-6879(99)99005-5

[22] Karatas F Karatepe M Baysar A Determination of free malondialdehyde in human serum by high-performance liquid chromatography Anal Biochem 2002 311 76 9 12441155 10.1016/s0003-2697(02)00387-1

[23] McConnell JD Bruskewitz R Walsh P Andriole G Lieber M Holtgrewe HL The effect of finasteride on the risk of acute urinary retention and the need for surgical treatment among men with benign prostatic hyperplasia N Engl J Med 1998 338 9 557 63 9475762 10.1056/NEJM199802263380901

[24] Roehrborn CG Boyle P Nickel JC Hoefner K Andriole G Efficacy and safety of a dual inhibitor of 5-alpha-reductase types 1 and 2 (dutasteride) in men with benign prostatic hyperplasia Urology 2002 60 3 434 41 12350480 10.1016/s0090-4295(02)01905-2

[25] Carson C Rittmaster R The role of dihydrotestosterone in benign prostatic hyperplasia Urology 2003 61 2 7 10.1016/s0090-4295(03)00045-1 12657354

[26] Glassman DT Chon JK Borkowski A Jacobs SC Kyprianou N Combined effect of terazosin and finasteride on apoptosis, cell proliferation, and transforming growth factor-β expression in benign prostatic hyperplasia Prostate 2001 46 1 45 51 11170131 10.1002/1097-0045(200101)46:1<45::aid-pros1007>3.0.co;2-u

[27] Kim HW Moon DG Kim HM Hwang JH Kim SC Nam SG Effect of shifting from combination therapy to monotherapy of α-blockers or 5α-reductase inhibitors on prostate volume and symptoms in patients with benign prostatic hyperplasia Korean J Urol 2011 52 681 6 22087362 10.4111/kju.2011.52.10.681 PMC3212662

[28] Wilt T MacDonald R Ishani A β-sitosterol for the treatment of benign prostatic hyperplasia: a systematic review BJU Int 1999 83 976 83 10368239 10.1046/j.1464-410x.1999.00026.x

[29] Caine M Alpha-adrenergic mechanisms in dynamics of benign prostatic hypertrophy Urology 1988 32 16 20 2462300

[30] Tóth I Szécsi M Julesz J Faredin L Behnke B In vitro inhibition of testicular Δ5-3β-hydroxysteroid dehydrogenase and prostatic 5α-reductase activities in rats and humans by strogen forte extract Int Urol Nephrol 1996 28 337 48 8899474 10.1007/BF02550496

[31] Hiipakka RA Zhang HZ Dai W Dai Q Liao S Structure–activity relationships for inhibition of human 5α-reductases by polyphenols Biochem Pharmacol 2002 63 1165 76 11931850 10.1016/s0006-2952(02)00848-1

[32] Roehrborn CG Malice MP Cook ThJ Girman CJ Clinical predictors of spontaneous acute urinary retention in men with LUTS and clinical BPH: a comprehensive analysis of the pooled placebo groups of several large clinical trials Urology 2001 58 210 6 11489703 10.1016/s0090-4295(01)01155-4

[33] Ren F Zhang Sh Mitchell SH Butler R Young CYF Tea polyphenols down-regulate the expression of the androgen receptor in LNCaP prostate cancer cells Oncogene 2000 19 1924 32 10773882 10.1038/sj.onc.1203511

[34] Gupta S Park A Shareef M Therapeutic effects of curcumin and thymoquinone in benign prostatic hyperplasia (BPH): role of inflammatory cytokines and chemokines Canc Res 2009 69 18 22

[35] Ragheb A Attia A Eldin WSh Elbarbry F Gazarin S Shoker A The protective effect of thymoquinone, an antioxidant and anti-inflammatory agent, against renal injury: a review Saudi J Kidney Dis Transpl 2009 20 741 52 19736468

[36] Jonas A Rosenblat G Krapf D Bitterman W Neemanet I Cactus flower extracts may prove beneficial in benign prostatic hyperplasia due to inhibition of 5α reductase activity, aromatase activity and lipid peroxidation Urol Res 1998 26 265 70 9760000 10.1007/s002400050055

[37] Hosseinzadeh H Parvardeh S Nassiri Asl M Sadeghnia HR Ziaee T Effect of thymoquinone and *Nigella sativa* seeds oil on lipid peroxidation level during global cerebral ischemiareperfusion injury in rat hippocampus Phytomedicine 2007 14 621 7 17291733 10.1016/j.phymed.2006.12.005

[38] Houcher Z Boudiaf Kh Benboubetra M Houcher B Effects of methanolic extract and commercial oil of *Nigella sativa L*. On blood glucose and antioxidant capacity in alloxaninduced diabetic rats Pteridines 2007 18 8 18

