# Supplementary material for: Inhibitory effects of Nigella sativa seed oil on the testosterone-induced benign prostatic hyperplasia in rats
Source: Biomedicine (Taipei). 2021 Mar 1;11(1):19–25. doi: 10.37796/2211-8039.1083 (PMC8823465; doi:10.37796/2211-8039.1083)
Supplement: Supplementary file 2 [file bmed-11-01-019-s002.pdf]

original_articlefinal.docx

WORD COUNT 3128

TIME SUBMITTED 23-MAY-2020 01:19AM

PAPER ID 59016506

Original article

Inhibitory effects of *Nigella sativa* seed oil on the testosterone-induced benign prostatic hyperplasia in rats

Abstract

Background: Benign prostatic hyperplasia (BPH) is the most prevalent disease of the prostate in the elderly men. Since *Nigella sativa* has been reported to show various pharmacological effects, this study was conducted to examine the effect of *N. sativa* seed oil on experimental BPH.

Methods: The oil was extracted using the cold-pressing method. Fifty rats were divided into five groups of 10 each as follows: Group 1 orally (p.o.) received normal saline; groups 2-5 were castrated and subcutaneously received 5 mg/kg testosterone propionate for four weeks. Group 2 received finasteride (0.5 mg/kg, p.o.), Group 3, namely, BPH model, underwent no further treatment. Groups 4 and 5 were treated with 400mg/kg and 800mg/kg *N. sativa* seed oil, respectively, for 28 days. All groups received repeated testosterone injections for the following four weeks after BPH induction. At the completion of the treatments, rats were sacrificed and the prostate tissues removed. Wet weight, prostatic volume (PV) and prostatic index (PI) were determined. Serum prostate-specific antigen (PSA), dihydrotestosterone (DHT), malondialdehyde (MDA) and antioxidant levels were determined.

Results: Our results showed that oral treatment with 400 and 800 mg/kg *N. sativa* oil led to a significant decrease in PI, PV, DHT concentration, PSA, and serum MDA level, and also significantly increased serum antioxidant capacity.

Conclusions: The study demonstrated that the oil seed exerted anti-BPH effects which may be associated with its antioxidant properties *in vivo*.

Keywords: Antioxidant level; Benign prostatic hyperplasia; Dihydrotestosterone; Malondialdehyde; *Nigella sativa* seed oil; Prostate-specific antigen.

Background

Benign prostatic hyperplasia (BPH) is the most prevalent age-related disease of the prostate gland for men [1, 2]. Its symptoms include urinary tract obstruction, frequent urination, urinary retention, decreased diameter of the urinary tube and pressure of urine flow, and dribbling at the end of urination [3, 4]. The disease is characterized by prostate gland enlargement due to hyperproliferation of cellular components such as mesenchymal cells. The most common drug treatments for BPH include the use of α-adrenergic antagonists, 5-α-reductase inhibitors and alternative therapies such as natural products [5].

Recent studies have shown the relationship between oxidative stress (OS) and BPH. As a measure of OS, the level of the lipid peroxidation indicator, *malondialdehyde* (MDA) increases in BPH patients while plasma antioxidants' are suppressed [6, 7]. This implies that antioxidant therapies might have potential application in the management of BPH.

Belonging to the plant family of Ranunculaceae, *Nigella sativa* is native to southwestern Asia, southern Europe, northern Africa and Iran [8, 9]. Mainly cultivated for its black seeds, *N. sativa* has extensive applications as spice and medicinal plant. The seeds are also rich sources of fixed oil, which is renowned for high level of unsaturated fatty acids such as oleic, linoleic and linolenic acid. Previous studies have further reported that these fatty acids can prevent the proliferation of prostate cells induced by testosterone and DHT. Additionally, they are capable to inhibiting the 5-α-reductase, an enzyme drug target of DHT which is known to metabolise testosterone to dihydrotestosteron [10-12]. *N. sativa* has further been demonstrated to exert antioxidant and anti-inflammatory properties [13, 14]. No evidence has yet been reported, however, on the use of this plant to treat BPH. This study was thus conducted to evaluate the effect of *N. sativa* seed oil on rat model of BPH.

Methods

Oil extraction

N. sativa seeds were purchased from Shahrekord Agricultural Faculty, Shahrekord, Iran. Sample identification was done by a botanical expert (Shirmardi, Hamzeh Ali, PhD.) at the Iranian Research Center of Agriculture and Natural Resources (P.O. Box 415, Shahrekord). Cultivation of *N. sativa* was performed in the region of (32° 21′ 00″ North, 50° 49′ 00″ East) where the average rainfall from cultivation to harvest is reportedly 5-7 mm. A voucher specimen (SKUMS-801) was approved and issued by the Herbarium of Medical Plants Research Center affiliated to Shahrekord University of Medical Sciences. The seeds were kept in polyethene bags at 4°C until extraction using screwless cold presses [15].

Experimental design

Fifty male Wistar rats weighing between 200 and 250 g were obtained from the Pasteur Institute of Iran (Tehran, Iran). The animals were housed under 21-23°C temperature and 12-h light and 12-h darkness cycles for seven days to acclimatize to the animal house. All stages of experimentation were carried out in accordance with the regulations of the ethic committee of shahrekord university of medical sciences (ethics code: IR.SKUMS.REC.1394.28).

Castration and testosterone-induced rat model of BPH

First, the rats were anaesthetized using 50 mg/kg phenobarbital and their testes were removed under sterile conditions. After castration, penicillin (7.14× 104 IU/kg body weight) was administrated intramuscularly according to a previously described method [2, 16]. Seven days later, the animals subcutaneously received 5 mg/kg testosterone propionate daily for four weeks. Simultaneously, group 1, as negative controls, were administered with normal saline alone, group 2 were considered BPH models, group 3 were administered with 0.5 mg/kg oral finasteride[1] and considered positive control, and groups 4 and 5 were orally treated with 400

and 800 mg/kg of the oil of *N. sativa* seed, respectively. The doses of *N. sativa* seed oil were selected according to previous studies [17, 18]. After 28 days, the rats were fasted for 24 hours and blood samples were drawn from the abdominal aorta under deep anesthesia. The prostate glands were then removed for further examinations. At the end of the experiments, rats were euthanized using high dose of co-administered ketamine and xylazine.

Determination of prostate index (PI) and volume (PV)

After anaesthesia, the whole prostates were collected and immediately weighed. The prostate weight/total body weight ratio was considered to indicate PI (Atawia et al., 2014) and the immersion of prostate in graded acetone was measured to indicate PV [19].

Measurement of prostate-specific antigen (PSA)

The PSA was measured by Biotin double antibody sandwich method by means of an ELISA kit (Shanghaicristal Day Biotech Co., China) according to the manufacturer's instructions.

Determination of dihydrotestosterone (DHT)

DHT was measured by mean of standard ELISA kits (Shanghaicristal Day Biotech Co., China) according to the manufacturer's instructions (Shanghaicristal Day Biotech Co., China). Competition occurred between an unlabeled antigen and enzyme-labelled antigen (conjugate) for a few number of antibody binding sites on the micro well plate. After removing unbound material by washing, the enzyme substrate was added. The enzymatic reaction was finally terminated by adding the stop solution. The optical absorbance of each well was read at 450 nm wavelength by using a microtiter plate reader. The intensity of the formed color was inversely correlated with the DHT concentration in the sample. A

collection of standards was applied to plot a standard curve to be used for measuring DHT concentrations in the samples and controls [20].

Determination antioxidant capacity of the serum

Blood samples were collected from all animals by means of cardiac puncture, and the serum was separated by centrifugation. The Ferric Reducing Ability of Plasma (FRAP) assay was applied for measuring the total serum antioxidant capacity. This method is based on the ability of the serum to reduce ferric-tripiridyltriazine (Fe^{3+}-TPTZ) to a ferrous form (Fe^{2+}), yielding a blue coloured complex (Fe^{2+}- TPTZ) with maximum optical absorbance at 593 nm [20].

Determination of serum MDA levels

For measuring serum MDA level, 0.5 g of thiobarbituric acid was dissolved in 80 ml 20% acetic acid and then the pH of the solution was set at 3.5 by using NaOH. The final volume of the assay was then adjusted to 100 ml by addition of 20% acetic acid. Then, 100 μl of the serum sample was dissolved in 2.5 ml of the working solution and 100 μl of 8.1% *sodium dodecyl sulfate* (SDS). The samples were left in a water bath containing boiling water for 1 hour and then cooled and centrifuged at 4000 rpm. The supernatant's optical absorbance was read at a wavelength of 523 nm [21].

Statistical analysis

The data were presented as mean ± standard error of measurement. Data analysis was performed by one-way ANOVA and Tukey's test using the version 7 of the GraphPad Prism software. Data were considered statistically significant at the level of $P < 0.05$.

Results

The Effect of *N. sativa* seed oil on PI and PV

As Figures 1 and 2 illustrate, the highest PI and PV are observed in the BPH model group and the lowest PI and PV levels in the control group. Oral treatment with 400 and 800 mg/kg of the oil of *N. sativa* seed significantly decreased the PI and PV when compared to the BPH model group ($p< 0.05$). There was no significant difference in the PI between *N. sativa* seed oil-treated groups and the finasteride-treated group, although the PI value in *N. sativa* seed oil-treated groups was lower than the finasteride-treated group. There was a significant difference in PV between *N. sativa* seed oil (400 mg/kg)-treated and finasteride-treated groups ($p< 0.05$), but there was not any significant difference in PV between *N. sativa* seed oil (800 mg/kg)-treated and finasteride-treated groups.

The effect of *N. sativa* seed oil on DHT and PSA concentrations

As shown in Figures 3 and 4, the highest concentrations of DHT and PSA were observed in the BPH model group while the lowest DHT and PSA concentrations were evident in the control group. Oral treatment with 400 and 800 mg/kg of the oil of *N. sativa* seed significantly decreased the DHT and PSA concentrations compared to the BPH model group. The level of DHT also decreased significantly and more markedly in both *N. sativa* seed oil-treated groups when compared to the finasteride-treated group. Moreover, the PSA concentrations in 800 mg/kg *N. sativa* seed oil-treated and finasteride-treated groups were significantly different.

The effect of *N. sativa* seed oil on serum antioxidant capacity and MDA levels

As Figure 5 illustrates, *N. sativa* seed oil and finasteride treatment led to a significant rise in the serum antioxidant capacity when compared to to the BPH model group ($p < 0.05$). The results showed that treatment with 400 and 800 mg/kg of the oil of *N. sativa* seed significantly increased the serum antioxidant capacity when compared to the finasteride receiving groups. Figure 6 illustrates the effect of oral treatment with *N. sativa* seed oil on MDA concentration. Treatment with 800 mg/kg *N. sativa* seed oil significantly decreased the MDA concentration in comparison to the BPH model group. In addition, 400 and 800 mg/kg *N. sativa* seed oil treatment led to a significant decrease in MDA concentration when compared to the finasteride-treated groups.

Discussion

The BPH is a non-malignant growth of the epithelial and stromal cells of the prostate gland. 5α-Reductase inhibitors and alpha-1-adrenergic antagonists are two main agents commonly used to treat BPH. 5α-Reductase is an essential enzyme that converts testosterone to dihydrotestosterone [22-24].

Finasteride is a classical 5α-reductase inhibitor that decreases the DTH level, resulting in a decrease in the PV and symptoms of patients with BPH [25, 26]. It has been well established that α_1-*adrenoreceptor* blockers relax prostatic smooth muscles thereby increasing urine flow while decreasing the prostate size and PSA [27]. The beneficial effects of medicinal plants in treating BPH have already been confirmed [8]. It has been well established that medicinal plants used to treat BPH decrease the plasma and prostate levels of the DHT and consequently suppress prostate weight and size [28]. In the current study and consistent with previous studies [3, 25, 27, 29], we observed that induction of BPH led to increase in PSA, DHT, PV, and PI in a rat model. Besides that, our findings showed that *N. sativa* seed oil treatment significantly mitigated these pathological markers.

In the current study, treatment with 400 and 800 mg/kg of the oil of *N. sativa* seed decreased the DHT level. Interestingly, the effect of this oil was more potent than that of finasteride with respect to decreasing the DHT level. Hiipakka et al. reported that treatment with polyphenols isolated from green tea decreased the DHT production, and inhibited prostate cells proliferation. Because *N. sativa* contains polyphenolic compounds [30], it can be argued that, at least, inhibition of 5α-reductase contributes to the beneficial effect of this plant [31]. The high amount of fatty acids of *N. sativa* oil has essential unsaturated fatty acids (about 1%

omega-3, 25% omega-9 and 58% omega-6) in abundance [10]. Abdel-Rahman et al. have argued that compounds rich in fatty acids could prevent prostate cells proliferation by lowering testosterone and DHT concentrations [11]. Liang et al. further demonstrated that fatty acids could inhibit 5α-reductase [12]. It has been shown that increased prostate weight could be considered a marker to diagnose BPH, while PI is often used to determine the progression of BPH [32]. In the present study, both 400 and 800 mg/kg doses of the oil of *N. sativa* seed decreased the PI in BPH. In addition, PV was significantly lower in *N. sativa* seed oil-treated than in the control groups. Oral treatment with 800 mg/kg of the oil of *N. sativa* seed decreased the PV in comparison to the BPH model group.

Recently, it has been demonstrated that PV and PSA concentrations can be used to predict prostate cells growth. In this regard, PSA can be considered as an alternative index for PV and as a marker to detect the risk of prostate carcinoma [33]. Hence, increased PSA level represents increased proliferation of prostate cells. In the current study, treatment with 400 and 800 mg/kg *N. sativa* seed oil significantly decreased the PSA concentration compared to the BPH model group. Besides that, treatment with 400 mg/kg *N. sativa* seed oil significantly decreased the PSA level. Ren et al. reported that polyphenols can suppress the level of expression of PSA genes [34]. *N. sativa*'s effect in decreasing the PSA may thus be related to the presence of polyphenols.

It has been determined that inflammation contributes to the pathophysiology of BPH because inflammatory factors such as monocyte chemotactic protein-1 are overexpressed. Hence the levels of interleukin 10 receptor subunit alpha (IL-10RA) and Interleukin 8 receptor, beta (IL-8RB) rise in the BPH [35]. According to the study by Ragheb et al., thymoquinone isolated from *N. sativa* has an anti-inflammatory property and can decrease the expression of the above-mentioned inflammatory factors. It my thus seems that *N. sativa* oil's effect can be to some extent attributed to the presence of thymoquinone in *N. sativa* and the anti-

inflammatory property of this plant [36]. According to the study of Jonas et al., the antioxidant activities of plants help regulate cell proliferation and control in BPH [37]. It has been demonstrated that an increase in the level of MDA, which occurs in BPH, is a marker of lipid peroxidation and/or tissues damage. Increased MDA level in BPH has also been reported to be due to OS [6]. Hence, treatment with antioxidants may decrease the level MDA and other pathological markers of BPH. In our study, *N. sativa* seed oil treatment decreased the serum MDA level, which is consistent with the study by Hosseinzadeh et al [38]. Houcher reported that the oral treatment with *N. sativa* extract can causean increase in the FRAP capacity [39], which is consistent with our results.

Therefore, it seems that *N. sativa* oil's effects to inhibit lipid peroxidation and probably its anti-BPH effects are due to the presence of antioxidant and free radical-inhibiting compounds. The results on the serum MDA levels in the current study further demonstrated that these variables increased in the BPH group in comparison to the control group. In addition, the serum MDA levels decreased significantly in *N. sativa* oil-treated groups in comparison to the BPH group, which indicates the protective effects of the compounds present in the oi.Therefore, *N. sativa* oil could increase the antioxidant capacity of serum and decrease the level of MDA. It seems that this decrease in MDA levels is associated with the antioxidant capacity of the plant.

Conclusion

According to the current study, *N. sativa* seed oil may have application in treating BPH by decreasing the concentrations of DHT and PSA, and PI and PV and exerting an antioxidant effect. Further studies are required to isolate and identify the active component(s) of the oil.

Figure legends:

Fig. 1 Effect of *Nigella sativa* seed oil on prostate index; negative control: Healthy rats, positive control: Rats with BPH administered with 0.5 mg/kg finasteride, BPH model: Rats with BPH, 400 mg/kg NS: Rats with BPH treated with 400 mg/kg *N. sativa* seed oil, 800 mg/kg NS: Rats with BPH treated with 800 mg/kg *N. sativa* seed oil; * represent significant difference when compared to the BPH group ($p < 0.05$).

Fig. 2 Effect of *Nigella sativa* seed oil on prostate volume; negative control: Healthy rats, positive control: Rats with BPH administered with 0.5 mg/kg finasteride, BPH model: Rats with BPH,400 mg/kg NS: Rats with BPH treated with 400 mg/kg *N. sativa* seed oil;, 800 mg/kg NS: Rats with BPH treated with 800 mg/kg *N. sativa* seed oil; * $p < 0.05$, ** $p < 0.01$ and *** $p < 0.001$ compared to the BPH group, # $p < 0.05$ compared with NS 400(mg/kg) group.

Fig. 3 Effect of *Nigella sativa* seed oil on dihydrotestosterone concentration; negative control: Healthy rats, positive control: Rats with benign prostatic hyperplasia (BPH) administered with 0.5 mg/kg finasteride, BPH model: Rats with BPH, 400 mg/kg NS: Rats with BPH treated with 400 mg/kg *N. sativa* seed oil, 800 mg/kg NS: Rats with BPH treated with 800 mg/kg *N. sativa* seed oil; *$p<0.05$, ** $p<0.01$ and ***$p<0.001$ compared to the BPH group, ###$p<0.001$ compared to the NS (400 and 800mg/kg) groups.

Fig. 4 Effect of *Nigella sativa* seed oil on prostate-specific antigen concentration; negative control: Healthy rats, positive control: Rats with benign prostatic hyperplasia (BPH) administered with 0.5 mg/kg finasteride, BPH model: Rats with BPH, 400 mg/kg NS: Rats with BPH treated with 400 mg/kg *N. sativa* seed oil, 800 mg/kg NS: Rats with BPH treated with 800 mg/kg *N. sativa* seed oil; *$p<0.05$ compared to the BPH group, #$p<0.05$ compared to the negative control group.

Fig. 5 Effect of *Nigella sativa* seed oil on serum antioxidant capacity; negative control: Healthy rats, positive control: Rats with benign prostatic hyperplasia (BPH) administered with 0.5 mg/kg finasteride, BPH model: Rats with BPH, 400 mg/kg NS: Rats with BPH treated with 400 mg/kg *N. sativa* seed oil, 800 mg/kg NS: Rats with BPH treated with 800 mg/kg *N. sativa* seed oil; *$p<0.05$, **$p<0.01$ and ***$p<0.001$ compared to the BPH group.

Fig. 6 Effect of *Nigella sativa* seed oil on malondialdehyde concentration; negative control: Healthy rats, positive control: Rats with benign prostatic hyperplasia (BPH) administered with 0.5 mg/kg finasteride, BPH model: Rats with BPH, 400 mg/kg NS: Rats with BPH treated with 400 mg/kg *N. sativa* seed oil, 800 mg/kg NS: Rats with BPH treated with 800 mg/kg *N. sativa* seed oil; ***$p<0.001$ compared to the BPH model.

original_articlefinal.docx

ORIGINALITY REPORT

25%

SIMILARITY INDEX

PRIMARY SOURCES

1	Daniel K. Afriyie, George A. Asare, K. Bugyei, Samuel Adjei, Jiu-mao Lin, Jun Peng, Zhen-feng Hong. "Treatment of benign prostatic hyperplasia with Croton membranaceus in an experimental animal model", Journal of Ethnopharmacology, 2014<br>Crossref	115 words — 3%
2	Arezo Sadeghimanesh, Valiallah Khalaji-Pirbalouty, Zahra Lorigooini, Mahmoud Rafieian-Kopaei, Akram Torki, Zahra Rabiei. "Phytochemical and neuroprotective evaluation of Citrus aurantium essential oil on cerebral ischemia and reperfusion", Bangladesh Journal of Pharmacology, 2018<br>Crossref	82 words — 2%
3	Shirin Asgharian, Mohammad Reza Hojjati, Mohsen Ahrari, Elham Bijad, Fatemeh Deris, Zahra Lorigooini. " and rutin, as its major compound: investigating their effect on spatial memory and passive avoidance memory in rats ", Pharmaceutical Biology, 2020<br>Crossref	70 words — 2%
4	ecmjournal.org<br>Internet	51 words — 2%
5	Maryam Paseban, Saeed Niazmand, Mohammad Soukhtanloo, Naser T. Meibodi et al. "The Therapeutic Effect of Nigella sativa Seed on Indomethacin-induced Gastric Ulcer in Rats", Current Nutrition & Food Science, 2020<br>Crossref	37 words — 1%
6	K.M Fararh, Y Atoji, Y Shimizu, T Takewaki. "Isulinotropic properties of Nigella sativa oil in Streptozotocin plus Nicotinamide diabetic hamster", Research in	32 words — 1%

Veterinary Science, 2002
Crossref

7 Akram Torki, Valiallah Khalaji-Pirbalouty, Zahra Lorigooini, Mahmoud Rafieian-Kopaei, Arezo Sadeghimanesh, Zahra Rabiei. "Anchusa italica extract: phytochemical and neuroprotective evaluation on global cerebral ischemia and reperfusion", Brazilian Journal of Pharmaceutical Sciences, 2018 — 30 words — 1%
Crossref

8 Masood Sadiq Butt, Muhammad Tauseef Sultan. " : Reduces the Risk of Various Maladies ", Critical Reviews in Food Science and Nutrition, 2010 — 29 words — 1%
Crossref

9 Maryam Paseban, Saeed Niazmand, Mohammad Soukhtanloo, Naser Tayyebi Meybodi. "The preventive effect of Nigella sativa seed on gastric ulcer induced by indomethacin in rat", Journal of Herbmed Pharmacology, 2020 — 26 words — 1%
Crossref

10 Abdel-Fattah Mohamed Abdel-Fattah, Kinzo Matsumoto, Hiroshi Watanabe. "Antinociceptive effects of Nigella sativa oil and its major component, thymoquinone, in mice", European Journal of Pharmacology, 2000 — 24 words — 1%
Crossref

11 Bahram Hosseinzadeh Samani, Hooman Gudarzi, Sajad Rostami, Zahra Lorigooini, Zahra Esmaeili, Fatemeh Jamshidi-kia. "Development and optimization of the new ultrasonic-infrared-vacuum dryer in drying Kelussia odoratissima and its comparison with conventional methods", Industrial Crops and Products, 2018 — 22 words — 1%
Crossref

12 Fallah Huseini, H., M. Amini, R. Mohtashami, M. E. Ghamarchehre, Z. Sadeqhi, S. Kianbakht, and A. Fallah Huseini. "Blood Pressure Lowering Effect of <i>Nigella sativa</i> L. Seed Oil in Healthy Volunteers: A Randomized, Double-Blind, Placebo-controlled Clinical Trial : BLOOD PRESSURE LOWERING EFFECT OF SEED OIL", Phytotherapy — 21 words — 1%

Research, 2013.
Crossref

13 Woo-Young Jeon, Ohn Soon Kim, Chang-Seob Seo, Seong Eun Jin, Jung-Ae Kim, Hyeun-Kyoo Shin, Yong-ung Kim, Mee-Young Lee. "Inhibitory effects of Ponciri Fructus on testosterone-induced benign prostatic hyperplasia in rats", BMC Complementary and Alternative Medicine, 2017
Crossref
20 words — 1%

14 Baioni, L.. "Stanniocalcin 1 affects redox status of swine granulosa cells", Regulatory Peptides, 20110607
Crossref
17 words — 1%

15 "Chemistry of Phytopotentials: Health, Energy and Environmental Perspectives", Springer Science and Business Media LLC, 2012
Crossref
16 words — < 1%

16 Zahra Tofighi, Fatemeh Alipour, Hormoz Hadavinia, Mohammad Abdollahi, Abbas Hadjiakhoondi, Narguess Yassa. " Effective antidiabetic and antioxidant fractions of extract and their constituents ", Pharmaceutical Biology, 2014
Crossref
16 words — < 1%

17 Jinmeng Zhao, Xinyi Niu, Jinjin Yu, Xin Xiao, Wenqi Li, Lulu Zang, Zhen Hu, Paul Siu-Po IP, Weifeng Li. "Poria cocos polysaccharides attenuated ox-LDL-induced inflammation and oxidative stress via ERK activated Nrf2/HO-1 signaling pathway and inhibited foam cell formation in VSMCs", International Immunopharmacology, 2020
Crossref
15 words — < 1%

18 Zahra Lorigooini, Negin Salimi, Amin Soltani, Hossein Amini-Khoei. "Implication of NMDA-NO pathway in the antidepressant-like effect of ellagic acid in male mice", Neuropeptides, 2019
Crossref
14 words — < 1%

19 Majid Shirani, Zahra Lorigooini, Arsham Pouriamofrad, Zahra Keivani Hafshejani. " In vitro effect of hydroalcoholic extract of on human ureteric contractions ", Journal of Renal Injury Prevention, 2019
14 words — < 1%

Crossref

20 www.researchsquare.com
Internet
12 words — < 1%

21 clok.uclan.ac.uk
Internet
11 words — < 1%

22 d-nb.info
Internet
11 words — < 1%

23 Mohammad Amin Sherafat, Abdolaziz Ronaghi, Leila Ahmad-Molaei, Mohammad Nejadhoseynian et al. "Kindling-induced learning deficiency and possible cellular and molecular involved mechanisms", Neurological Sciences, 2012
Crossref
10 words — < 1%

24 aak.com
Internet
10 words — < 1%

25 baadalsg.inflibnet.ac.in
Internet
9 words — < 1%

26 Malhotra, S. K.. "Nigella", Handbook of herbs and spices, 2012.
Crossref
8 words — < 1%

27 Salem, M.L.. "Immunomodulatory and therapeutic properties of the Nigella sativa L. seed", International Immunopharmacology, 200512
Crossref
8 words — < 1%

28 link.springer.com
Internet
8 words — < 1%

29 Edible Medicinal And Non-Medicinal Plants, 2013.
Crossref
8 words — < 1%

30 V. Tembhurne S., Feroz S., H. More B., M.Sakarkar D.. "A review on therapeutic potential of Nigella sativa (kalonji) seeds", Journal of Medicinal Plants Research, 2014
Crossref
8 words — < 1%

31 Jing Shi, Lan Wang, Yan Lu, Yue Ji, Yaqing Wang, Ke Dong, Xiangqing Kong, Wei Sun. "Protective effects of seabuckthorn pulp and seed oils against radiation-induced acute intestinal injury", Journal of Radiation Research, 2017 — 8 words — < 1%
Crossref

32 Abolfazl Dadkhah, Faezeh Fatemi, Somaieh Kazemnejad, Yusef Rasmi, Javad Ashrafi-Helan, Abdolamir Allameh. "Differential effects of acetaminophen on enzymatic and non-enzymatic antioxidant factors and plasma total antioxidant capacity in developing and adult rats", Molecular and Cellular Biochemistry, 2006 — 8 words — < 1%
Crossref

33 Iori Sakakibara, Takahiro Fujino, Makoto Ishii, Toshiya Tanaka et al. "Fasting-Induced Hypothermia and Reduced Energy Production in Mice Lacking Acetyl-CoA Synthetase 2", Cell Metabolism, 2009 — 8 words — < 1%
Crossref

34 www.ncbi.nlm.nih.gov — 8 words — < 1%
Internet

35 Mohammad Rahimi-Madiseh, Azar Naimi, Esfandiar Heydarian, Mahmoud Rafieian-Kopaei. "Renal biochemical and histopathological alterations of diabetic rats under treatment with hydro alcoholic Morus nigra extrac", Journal of Renal Injury Prevention, 2016 — 8 words — < 1%
Crossref

36 Mohammad Reza Khazdair. " The Protective Effects of and Its Constituents on Induced Neurotoxicity ", Journal of Toxicology, 2015 — 8 words — < 1%
Crossref

37 Mastour S. Al-Ghamdi. " Protective Effect of Seeds Against Carbon Tetrachloride-induced Liver Damage ", The American Journal of Chinese Medicine, 2012 — 6 words — < 1%
Crossref

38	T. K. Lim. "Edible Medicinal And Non-Medicinal Plants", Springer Science and Business Media LLC, 2013<br>Crossref	6 words — < 1%
39	I. Meral. "Effect of Nigella sativa on Glucose Concentration, Lipid Peroxidation, Anti-Oxidant Defence System and Liver Damage in Experimentally-Induced Diabetic Rabbits", Journal of Veterinary Medicine Series A, 12/9/2001<br>Crossref	6 words — < 1%

EXCLUDE QUOTES	OFF	EXCLUDE MATCHES	OFF
EXCLUDE BIBLIOGRAPHY	OFF		
