# Supplementary material for: Inhibitory effects of Nigella sativa seed oil on the testosterone-induced benign prostatic hyperplasia in rats
Source: Biomedicine (Taipei). 2021 Mar 1;11(1):19–25. doi: 10.37796/2211-8039.1083 (PMC8823465; doi:10.37796/2211-8039.1083)
Supplement: Supplementary file 4 [file bmed-11-01-019-s004.docx]

# Authors’ Response to the Reviewer's Comments

***Journal:***  BioMedicine

***Title of Paper: Inhibitory effects of Nigella sativa seed oil on the testosterone-induced benign prostatic hyperplasia in rats***

***Authors*: *Arezo Sadeghimanesh, Sajedeh Gholipour, Akram Torki, Hossein Amini-khoei, Zahra Lorigooini, and Solomon Habtemariam***

***Date Sent:***  28 July 2020

We appreciate the time and efforts by the reviewer for careful and thorough reading of this manuscript and for the thoughtful comments and constructive suggestions, which help to improve the quality of this manuscript. We believed that the revised version can meet the journal publication requirements. Our response follows (the reviewer’s comments are in italics).

**Reviwer 1:**

*GENERAL COMMENTS: This is an interesting study, as authors tried assessing the efficacy of Nigella sativa seed oil on the testosterone-induced benign prostatic hyperplasia in rats as a potential alternative therapy. Nevertheless, there are issues that need clarification. Below are some of them. Also, Authors proofread the manuscript thoroughly to rid it of typo and grammatical errors. ABSTRACT: Authors wrote that ''All groups received repeated testosterone injections for the following four weeks after BPH induction''. Was this done during the treatment period? So at what point was the condition (BPH) established and treated? Authors should come clear on this. INTRODUCTION: Authors should justify with reasons why an alternative therapy is needed for BPH, and why natural products (Plant products) are the best alternative. METHODS:*

Thanks for the insightful comment, it was revised.

**BPH induction with received repeated testosterone injections**

**treated with *N*. *sativa* seed oil**

**Acclimatizing**

day 0 day 7 day 28

**Comment 1:**

*1. Oil extraction: The sentence ''Cultivation of N. sativa was performed in the region of (32° 21′ 00″ North, 50° 49′ 00″ East) where the average rainfall from cultivation to harvest is reportedly 5-7 mm'' on line 4 is not clear. Does it mean after purchasing the seeds they were cultivated again before they were used for the study. It is an ambiguous statement and needs clarification. Also, the title 'Oil extraction' does not fit the content. Authors did not describe any extraction methodology under this section to warrant such a title.*

Thanks for the insightful comment, we were revised and rewritten this part. Also, it should be mentioned that the cultivated seed plant was purchased, so in this part we brought the cultivation of condition.

**Comment 2:** *Experimental design: Again the title does not fit the content. There is no description of the study design in this section. It only talks about how the experimental animals were procured and housed.*

Thanks for the insightful comment, it was improved.

**Comment 3:** *Castration and testosterone-induced rat model of BPH: How relevant was it to castrate the animals before the testosterone-induction. Also, how did Authors determine a successful establishment of the BPH model before treatment. This dhould be stated clearly in the manuscript because the study is all about this. In the abstract section, Authors indicated that induction continued during the 4 weeks of treatment, but this statement is missing in this section. Authors should come clear on this. If this was the case, it will definitely had affected the study outcome.*

Thanks for the insightful comment, it was improved and added. It has been shown in previous studies that induction of BPH causes urinary retention in the bladder, which can be detected by tapping the lower abdomen of mice.

**Comment 4:**
*Determination of prostate index (PI) and volume (PV): Authors should write clearly the formulae used to determine PI and PV. They should stand out.*

Thanks for the insightful comment, *prostate index (PI) formulae* was added and in our study The method of calculating PV has no formula.

**Comment 5:**

*Determination of dihydrotestosterone (DHT): Authors should describe the ELISA method used with appropriate figures. The description given is not adequate and inappropriate. RESULTS:*

Thanks for the insightful comment, it was revised.

**Comment 6:**

*The Effect of N. sativa seed oil on PI and PV: Authors should explain the Figure 2. The level of significance indicated are not appropriate, especially the positive control, NS 400 and NS 800. Also, it seems there was no significant effect among treatment groups. Please, justify with relevant figures. DISCUSSION: Authors cannot make this conclusion when different doses were used in both instances ''Interestingly, the effect of this oil was more potent than that of finasteride with respect to decreasing the DHT level''. ''Interestingly, the effect of this oil was more potent than that of finasteride with respect to decreasing the DHT level''*

Thanks for the insightful comment, it was revised. We double checked our results and we observed that but 400 and 800 mg/kg of *N*. *sativa* oil significantly reduced the negative effect of BPH model in our various tests. However, we did not observe any significant changes between dose of 400 and dose of 800 mg/kg of *N*. *sativa* oil in our experiments.

**Reviwer 2:**

*I have reviewed the manuscript and here are my comments:*

**Comment 1:**

*the manuscript is generally well written and has novel and scientifically*

*important results.*

Thanks for the insightful comment.

**Comment 2:**

*Groups 2 and 3 have been interchanged in the abstract, group 2 should be BPH.*

Thanks for the insightful comment, it was improved.

**Comment 3:**

*I could not find the figures, however, the authors did not mention in the results the presence of dose dependent effect between the two doses used.*

Thanks for the insightful comment, it was corrected. Both doses of the 400 and 800 mg/kg showed this effective effect.

**Comment 4:**

*the authors should address the issue of dose dependent effect above n the discussion as well.*

Thanks for the insightful comment, it was corrected. Both doses of the 400 and 800 mg/kg showed this effective effect.

**Comment 5:**

*There are several typographical errors.*

Thanks for the insightful comment, it was corrected.
