# Supplementary material for: Inhibitory effects of Nigella sativa seed oil on the testosterone-induced benign prostatic hyperplasia in rats
Source: Biomedicine (Taipei). 2021 Mar 1;11(1):19–25. doi: 10.37796/2211-8039.1083 (PMC8823465; doi:10.37796/2211-8039.1083)
Supplement: Supplementary file 5 [file bmed-11-01-019-s005.docx]

**Figure 1**. Schematic of study design


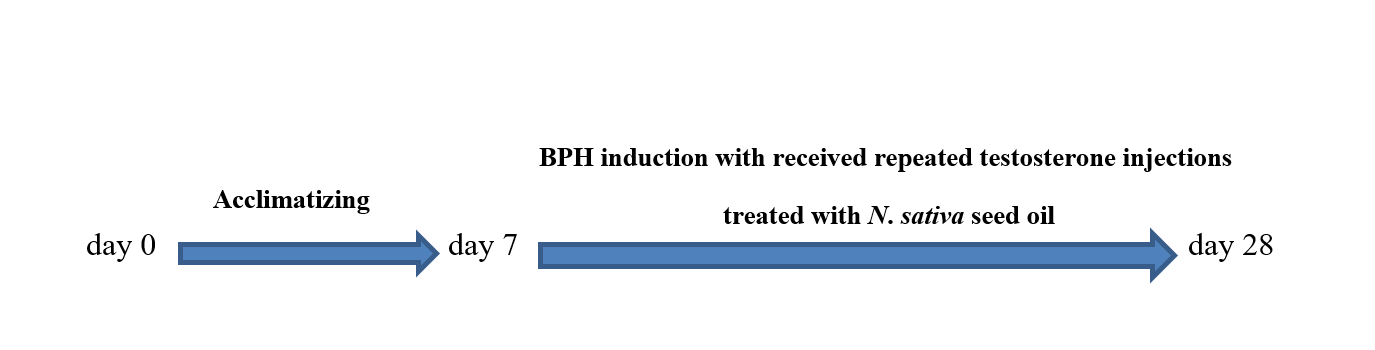


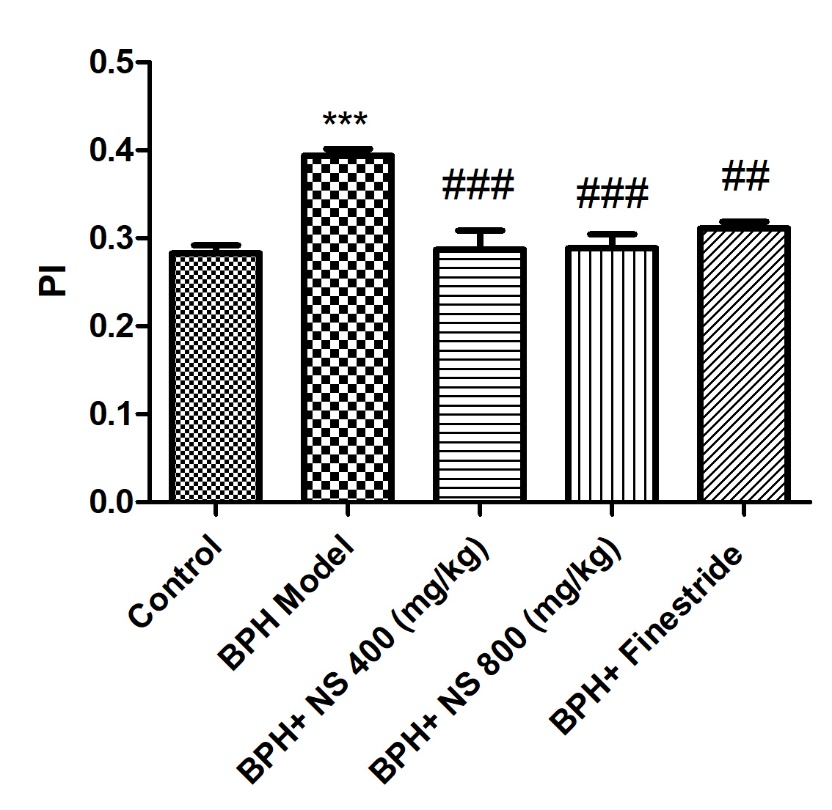


**Figure 2**. Effect of *Nigella sativa* seed oil on prostate index; control: Healthy rats, BPH model: Rats with BPH, BPH+ NS 400 (mg/kg): Rats with BPH treated with 400 mg/kg *N*. *sativa* seed oil, BPH+ NS 800 (mg/kg): Rats with BPH treated with 800 mg/kg *N*. *sativa* seed oil, BPH+Finestride: Rats with BPH administered with 0.5 mg/kg finasteride; *** significant difference with control group (*p*< 0.001), ###,###,## significant difference with BPH group (*p*< 0.001, *p*< 0.001, *p*< 0.01).


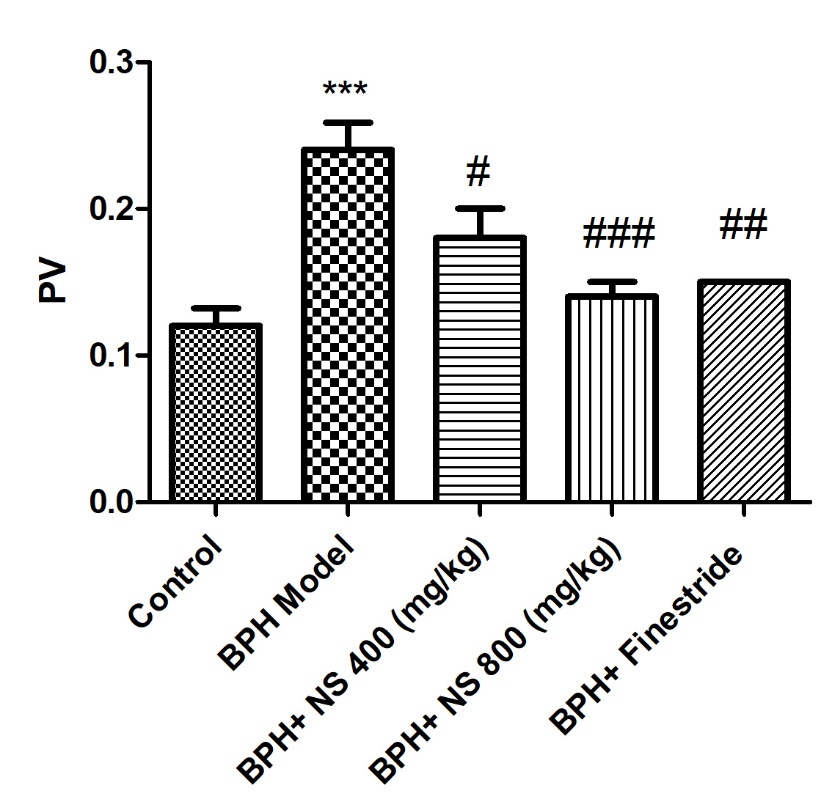


**Figure 3**. Effect of *Nigella sativa* seed oil on prostate volume; control: Healthy rats, BPH model: Rats with BPH, BPH+ NS 400 (mg/kg): Rats with BPH treated with 400 mg/kg *N*. *sativa* seed oil, BPH+ NS 800 (mg/kg): Rats with BPH treated with 800 mg/kg *N*. *sativa* seed oil, BPH+Finestride: Rats with BPH administered with 0.5 mg/kg finasteride; *** significant difference with control group (*p*< 0.001), #, ###, ## significant difference with BPH model group (*p*< 0.05, *p*< 0.001, *p*< 0. 01).


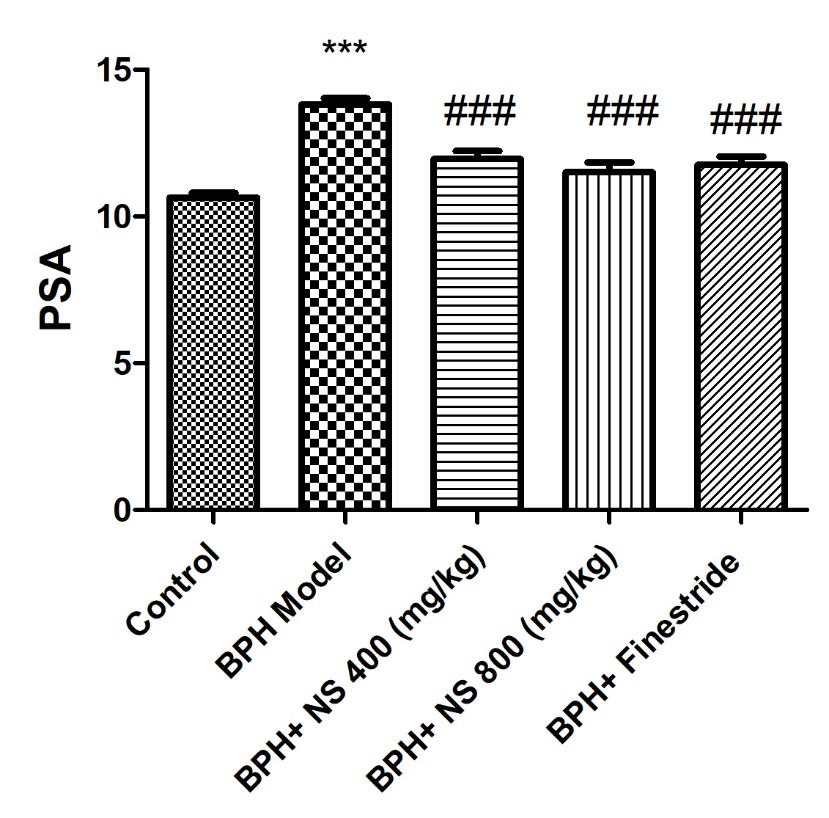


**Figure 4.** Effect of *Nigella sativa* seed oil on dihydrotestosterone concentration; control: Healthy rats, BPH model: Rats with BPH, BPH+ NS 400 (mg/kg): Rats with BPH treated with 400 mg/kg *N*. *sativa* seed oil, BPH+ NS 800 (mg/kg): Rats with BPH treated with 800 mg/kg *N*. *sativa* seed oil, BPH+Finestride: Rats with BPH administered with 0.5 mg/kg finasteride; *** significant difference with control group (*p*< 0.001), ### significant difference with BPH model group (*p*< 0.001).


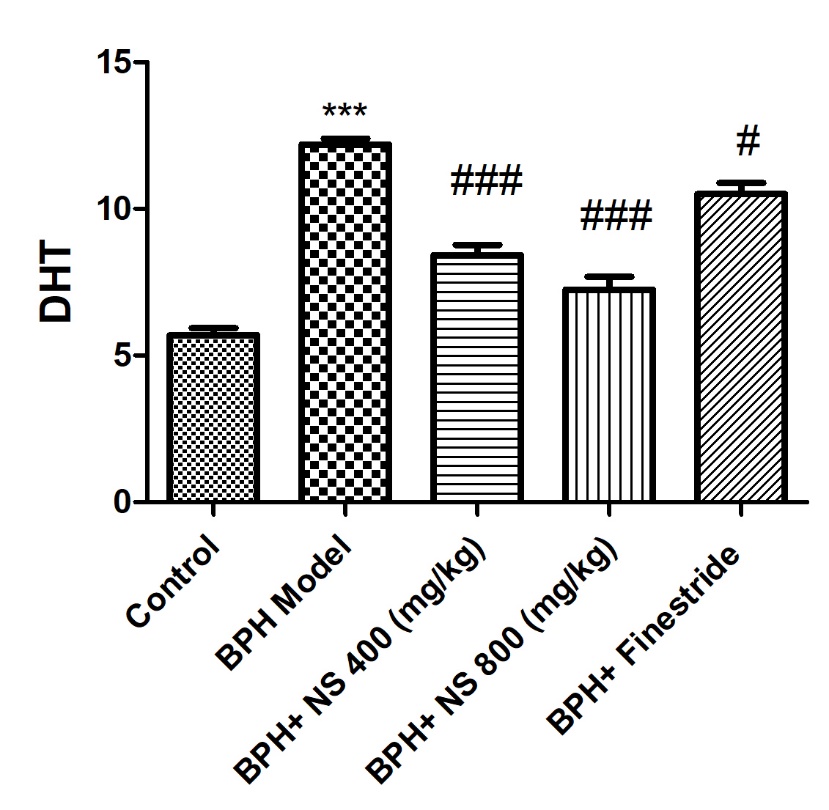


**Figure 5.** Effect of *Nigella sativa* seed oil on prostate-specific antigen concentration; control: Healthy rats, BPH model: Rats with BPH, BPH+ NS 400 (mg/kg): Rats with BPH treated with 400 mg/kg *N*. *sativa* seed oil, BPH+ NS 800 (mg/kg): Rats with BPH treated with 800 mg/kg *N*. *sativa* seed oil, BPH+Finestride: Rats with BPH administered with 0.5 mg/kg finasteride; *** significant difference with control group (*p*< 0.001), ###, ###, # significant difference with BPH model group (*p*< 0.001, *p*< 0.001, *p*< 0.05).


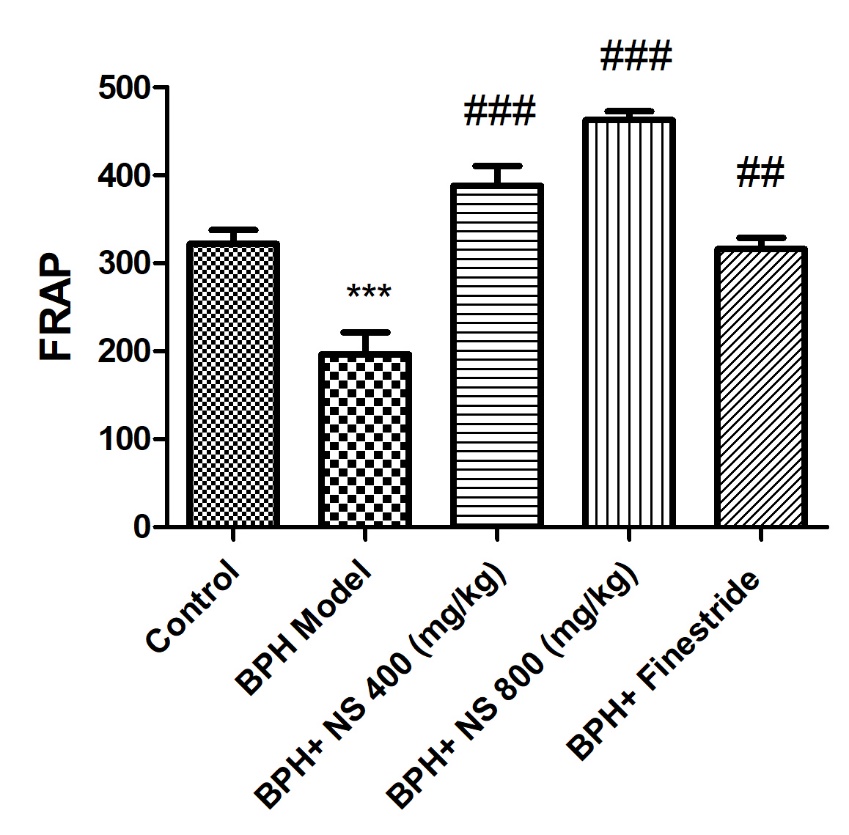


**Figure 6.** Effect of *Nigella sativa* seed oil on serum antioxidant capacity; control: Healthy rats, BPH model: Rats with BPH, BPH+ NS 400 (mg/kg): Rats with BPH treated with 400 mg/kg *N*. *sativa* seed oil, BPH+ NS 800 (mg/kg): Rats with BPH treated with 800 mg/kg *N*. *sativa* seed oil, BPH+Finestride: Rats with BPH administered with 0.5 mg/kg finasteride; *** significant difference with control group (*p*< 0.001), ###, ###, ## significant difference with BPH model group (*p*< 0.001, *p*< 0.001, *p*< 0.01).


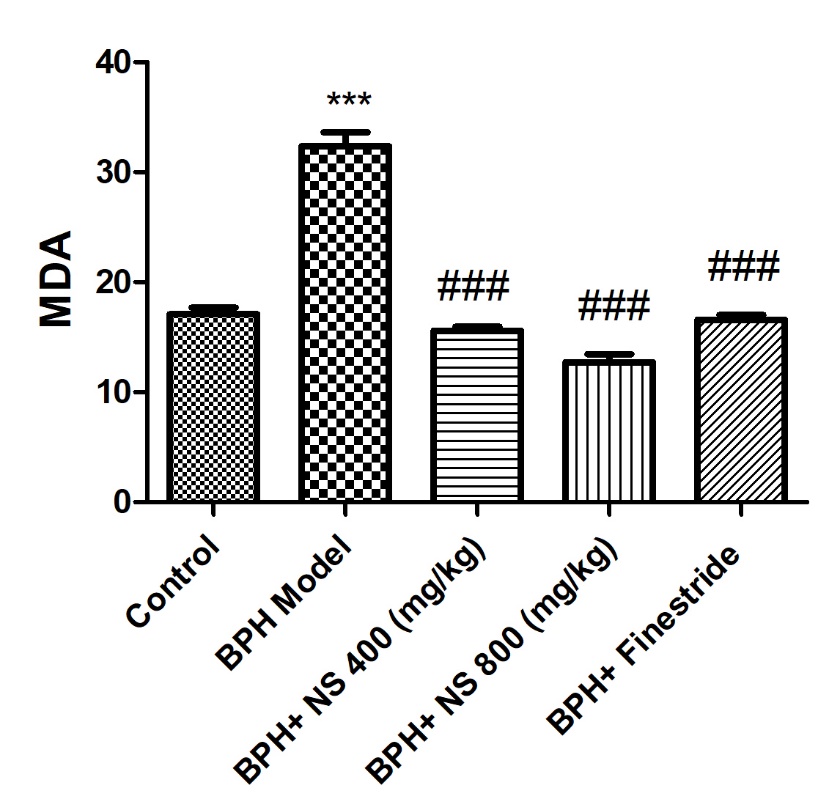


**Figure 7.** Effect of *Nigella sativa* seed oil on malondialdehyde concentration; control: Healthy rats, BPH model: Rats with BPH, BPH+ NS 400 (mg/kg): Rats with BPH treated with 400 mg/kg *N*. *sativa* seed oil, BPH+ NS 800 (mg/kg): Rats with BPH treated with 800 mg/kg *N*. *sativa* seed oil, BPH+Finestride: Rats with BPH administered with 0.5 mg/kg finasteride; *** significant difference between control group and other groups (*p*< 0.001), ### significant difference with BPH model group (*p*< 0.001).
